# Harnessing *Pseudomonas* spp. for sustainable plant crop protection

**DOI:** 10.3389/fmicb.2024.1485197

**Published:** 2024-11-21

**Authors:** Hussain Alattas, Bernard R. Glick, Daniel V. Murphy, Colin Scott

**Affiliations:** ^1^Bioplastics Innovation Hub, Food Futures Institute, Murdoch University, Murdoch, WA, Australia; ^2^School of Medical, Molecular, and Forensic Sciences, Murdoch University, Murdoch, WA, Australia; ^3^Department of Biology, University of Waterloo, Waterloo, ON, Canada; ^4^SoilsWest, Centre for Sustainable Farming Systems, Food Futures Institute, Murdoch University, Murdoch, WA, Australia; ^5^CSIRO Environment, Black Mountain Science and Innovation Park, Canberra, ACT, Australia

**Keywords:** biocontrol, sustainable agriculture, plant-microbe interactions, rhizosphere, crop protection, phytopathogen suppression

## Abstract

This review examines the role of *Pseudomonas* spp. bacteria as biocontrol agents against crop diseases, focusing on their mechanisms of action, efficacy, and potential applications in sustainable agriculture. *Pseudomonas* spp., ubiquitous in soil ecosystems and root microbiomes, have attracted attention for their ability to suppress phytopathogens and enhance plant health through various mechanisms. These include direct competition for nutrients, production of antimicrobial compounds and volatile organic compounds, competition using type VI secretion systems, and indirect induction of systemic resistance. Our review shows that *Pseudomonas* strains effectively control a wide range of diseases across diverse plant species, with some strains demonstrating efficacy comparable to chemical fungicides. However, the review also highlights challenges in achieving consistent performance when using *Pseudomonas* inoculants under field conditions due to various biotic and abiotic factors. Strategies to optimize biocontrol potential, such as formulation techniques, application methods, and integration with other management practices, are discussed. The advantages of *Pseudomonas*-based biocontrol for sustainable agriculture include reduced reliance on chemical pesticides, enhanced crop productivity, and improved environmental sustainability. Future research directions should focus on understanding the complex interactions within the plant microbiome, optimizing delivery systems, and addressing regulatory hurdles for commercial deployment. This review underscores the significant potential of *Pseudomonas* spp. in sustainable crop protection while acknowledging the need for further research to fully harness their capabilities in agricultural systems.

## Graphical abstract

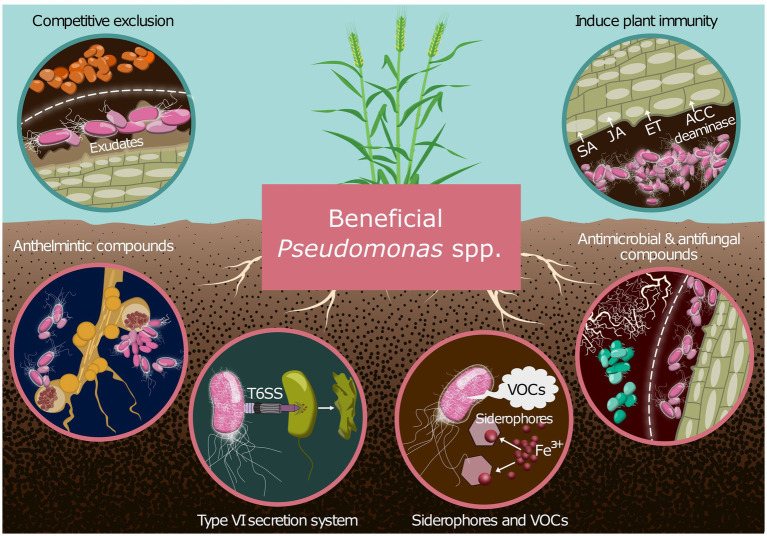

## 1 Introduction

The UN’s Sustainable Development Goals, released in 2015, outline 17 urgent objectives to be achieved through global collaboration. Among these, Goal 2 aims to end hunger, achieve worldwide food security, improve nutrition, and promote sustainable agriculture. As the world’s population increases, the demand for food production also rises. This increases the pressure on agricultural systems globally (United Nations, 2015). One important development that will help address these issues is the growing popularity in some countries of plant-based diets as an alternative to traditional meat and dairy consumption.

Chemical fertilizers play a crucial role in meeting the increasing demand for plant-based food by maximizing crop yields (Bhatti et al., 2017; Hera, 1995; Pahalvi et al., 2021). The three primary nutrients in commercial fertilizers, nitrogen (N), phosphate (P), and potassium (K) are used extensively in modern agriculture (McGuire, 2015). However, these fertilizers can also disrupt natural soil processes, leading to reduced water retention, imbalanced soil fertility, declined the agricultural soil quality with the reduction in soil organic matter (Dar et al., 2016; Dinesh et al., 2010; Ongley et al., 2010).

Similarly, the widespread use of pesticides to combat crop diseases poses significant environmental and health risks. Many pesticides are toxic to humans, animals, and non-target organisms, including essential pollinators (Brevik and Burgess, 2012; Geiger et al., 2010; Lee and Choi, 2020; Sponsler et al., 2019). The ecological impacts of many pesticides extend to soil and water systems. These chemicals disrupt microbial communities and reduce soil fertility (Law et al., 2017; Pimentel et al., 1993; Viaene et al., 2016). The loss of beneficial microbial species in the agricultural soil can exacerbate pathogen invasions and compromise ecosystem resilience (Jacobsen and Hjelmsø, 2014; Meena et al., 2020). Since microbial communities are the primary drivers of soil nutrient cycling, due to their various metabolic activities, their relevance in moderating ecosystem function cannot be understated (Balser et al., 2002).

In response to these challenges, there is a growing imperative to explore sustainable alternatives to conventional agricultural practices (Vasilescu et al., 2023). In this regard, microbiological tools, such as biofertilizers and biocontrol agents, have emerged as promising solutions. Biofertilizers, containing beneficial microorganisms, enhance soil fertility and promote plant growth through natural processes (Verma et al., 2019). Biocontrol agents offer non-chemical methods for managing plant diseases by leveraging the antagonistic properties of microorganisms against pathogens (Bhardwaj et al., 2014; Bonaterra et al., 2022; Parani and Saha, 2012).

The bacterial genus *Pseudomonas*, characterized by its metabolic diversity and abundance in various environments, has garnered particular attention for its potential applications in agriculture (Palleroni, 2015). Members of this genus show considerable metabolic and genetic diversity (Peix et al., 2009); its various secondary metabolites are known to form virulence factors, pigments, and biofilms (Drenkard and Ausubel, 2002; Moissenet and Khedher, 2011; Raio and Puopolo, 2021; Stover et al., 2000). A large number of *Pseudomonas* strains are known for plant growth-promoting potential by producing various substances such as siderophores, 1-aminocyclopropane-1-carboxylate (ACC) deaminase and lipopeptides (Leontidou et al., 2020; Pršić and Ongena, 2020). Using microbes such as *Pseudomonas* offers a promising approach to sustainable farming, offering solutions that support plant health while minimizing environmental impacts (Hamid et al., 2021; Khatoon et al., 2020; Nikel et al., 2014; Sharma and Archana, 2016).

Here, we provide a comprehensive overview of *Pseudomonas* spp. as microbiological tools for sustainable agriculture, examining their status and potential applications in crop production, particularly in cereal crops. We discuss the mechanisms by which *Pseudomonas* spp. act as biocontrol agents, including direct inhibition of phytopathogens and induction of systemic resistance in plants. Additionally, we explore approaches for optimizing the efficacy of *Pseudomonas*-based biocontrol strategies, such as improved delivery methods and genetic engineering. Finally, we address the challenges and future directions in harnessing *Pseudomonas* for sustainable crop protection, emphasizing the importance of ongoing research in advancing environmentally friendly agricultural practices.

## 2 Diversity and plant interactions of *Pseudomonas* species

Key interactions between plants and microbes occur in the soil surrounding plant roots (Berg et al., 2014; Gaiero et al., 2013; Jain et al., 2020). The rhizosphere, the immediate soil layer influenced by root exudates, is a hotspot for microbial colonization, fostering diverse communities of bacteria, fungi, and other microorganisms (Berg et al., 2014; Gaiero et al., 2013; Jain et al., 2020). *Pseudomonas* spp. are often major components of the rhizosphere microbiome and engage in multifaceted interactions with plants, exerting significant influence on plant health, nutrient cycling, and ecosystem functioning (Botelho and Mendonça-Hagler, 2006; Chaudhary et al., 2021; Raio and Puopolo, 2021).

*Pseudomonas* spp. display remarkable adaptability to diverse environmental conditions, thriving in various soil types and agricultural settings (Palleroni, 2015). Their metabolic versatility and genetic plasticity enable them to colonize plant roots and establish intricate symbiotic relationships with their host plants (Palleroni, 2015; Peix et al., 2009). Many studies have demonstrated the enrichment of *Pseudomonas* spp. in the roots and rhizosphere of diverse plants such as *Arabidopsis thaliana*, potatoes, rice, wheat, and barley (Andreote et al., 2009; Buddrus-Schiemann et al., 2010; Jain et al., 2020; Lawongsa et al., 2008; Persello-Cartieaux et al., 2001; Raio and Puopolo, 2021; Wang et al., 2012). The adaptability of *Pseudomonas* spp. to different plant environments might have evolved due to selective pressures, leading to the development of metabolic pathways conducive to nutrient acquisition from plant-derived compounds (Imperato et al., 2019; Rainey, 1999).

At the molecular level, plant-*Pseudomonas* interactions are orchestrated through intricate signaling pathways and molecular dialogs (Girard et al., 2020; Preston, 2004). Root exudates, comprising a diverse array of largely low molecular weight, organic compounds serve as chemoattractants, guiding *Pseudomonas* migration toward the rhizosphere (Drigo et al., 2009; Jain et al., 2020; Marilley et al., 1999; Waldon et al., 1989; Wang et al., 2017). Upon encountering plant roots, *Pseudomonas* spp. employ chemotaxis and quorum sensing systems to modulate their behavior and adapt to changing environmental cues (Loh et al., 2002; Schikora et al., 2016).

**Figure 1:**
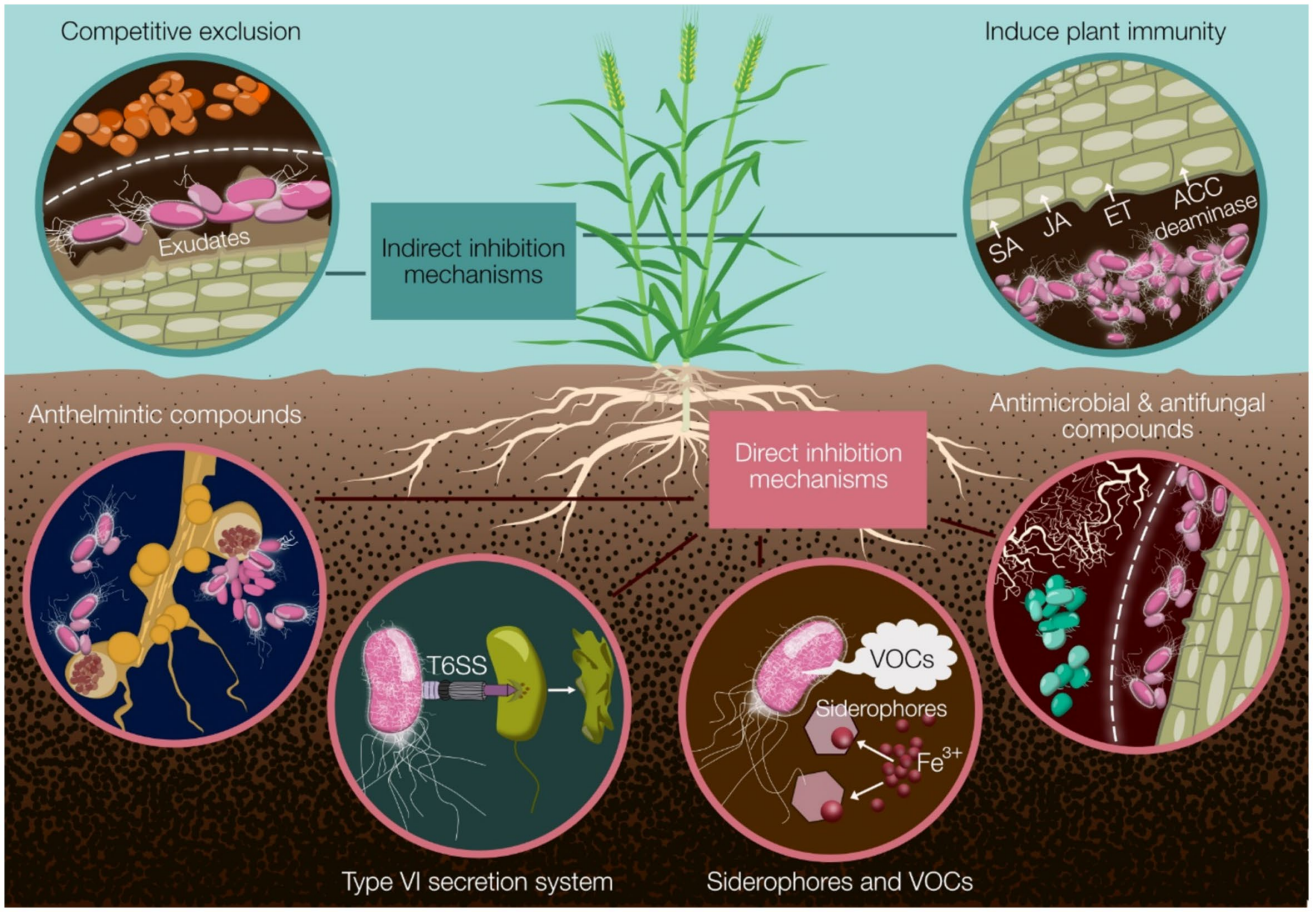
A summary of the major mechanisms by which *Pseudomonas* biocontrol strains can protect cereal crops from disease. Indirect mechanisms include competitive exclusion and induction of plant immunity through phytohormone modulation (SA, JA, ET) and ACC deaminase activity. Direct mechanisms encompass the production of anthelmintic, antimicrobial, and antifungal compounds; deployment of the type VI secretion system (T6SS); and secretion of siderophores and volatile organic compounds (VOCs). These diverse strategies collectively contribute to effective disease suppression and improved crop health. SA, salicylic acid; JA, jasmonic acid; ET, ethylene; ACC, 1-aminocyclopropane-1-carboxylate; T6SS, type VI secretion system; VOCs, volatile organic compounds.

*Pseudomonas* species actively participate in nutrient cycling processes, facilitating the uptake and assimilation of essential nutrients by plants (Botelho and Mendonça-Hagler, 2006; Chaudhary et al., 2021; Raio and Puopolo, 2021). Through the production of plant growth-promoting substances, such as phytohormones and siderophores (Leontidou et al., 2020), *Pseudomonas* species stimulate root growth, enhance nutrient acquisition, and confer resistance to environmental stresses (Raio and Puopolo, 2021; Sun et al., 2022). Moreover, *Pseudomonas* spp. produce a wide range of antimicrobial secondary metabolites. This diversity enables them to outcompete other microorganisms for niche space and carbon resources provided by plants (Figure 1) (Raio and Puopolo, 2021; Validov et al., 2005).

In addition to promoting plant growth and nutrient acquisition, *Pseudomonas* spp. play a pivotal role in suppressing plant pathogens and mitigating disease incidence (Hernández-León et al., 2015; Lahlali et al., 2022; Validov et al., 2005; Vivekananthan et al., 2004). Through the production of antimicrobial compounds, lytic enzymes, and volatile organic compounds, *Pseudomonas* spp. inhibit the proliferation of phytopathogens and confer protection to their host plants (Hernández-León et al., 2015; Mehmood et al., 2023; Raio and Puopolo, 2021; Validov et al., 2005; Vivekananthan et al., 2004). Furthermore, *Pseudomonas*-mediated induction of systemic resistance within host plants primes the plants for enhanced defense responses, bolstering their resilience against pathogen attacks (Haney et al., 2018; Leeman et al., 1995).

These attributes position *Pseudomonas* spp. as promising candidates for biocontrol agents in agriculture, as they not only contribute to plant protection but also promote plant growth under various environmental conditions, including high salinity and drought (Cheng et al., 2007; Forni et al., 2017; Orozco-Mosqueda et al., 2019; Rangarajan et al., 2003). However, it is essential to acknowledge that while many *Pseudomonas* strains act as beneficial or passive colonizers of the plant microbiome, certain species may exhibit phytopathogenic traits (Leontidou et al., 2020; Montes-Osuna et al., 2022; Pršić and Ongena, 2020; Young, 1991). One well-documented example is *Pseudomonas syringae*, known for causing various plant diseases by producing virulence factors such as coronafacic acid, ice nucleation protein, and the type III secretion system (T3SS) and its associated type III secreted effector (T3SE) proteins (Alattas et al., 2023; Bundalovic-Torma et al., 2022; Büttner, 2016; Weiler et al., 1994; Xin et al., 2018).

## 3 *Pseudomonas* as a plant pathogen

*P. syringae* is a highly diverse bacterial species complex known for its ability to cause disease in a wide range of plant hosts. The complex is currently divided into at least 13 phylogenetic groups or phylogroups, with 7 considered primary phylogroups that contain most of the recognized pathogenic strains (Dillon et al., 2021). Although strains are often classified into pathovars based on the host they were isolated from, there is not always a strong correlation between phylogeny and host specificity. Many closely related strains can infect different hosts, while strains isolated from the same host may be phylogenetically diverse (Baltrus et al., 2017; Morris et al., 2019).

Key factors in *P. syringae* pathogenicity are the T3SS and T3SE proteins. The T3SS protein allows the bacteria to inject effector proteins directly into plant cells, where they can manipulate host processes to promote infection (Büttner, 2016). *P. syringae* strains typically possess 20–30 different T3SE proteins, with over 70 distinct T3SE families identified across the species complex (Dillon et al., 2019). However, only a small number of core effectors are conserved across most strains. The diversity and composition of effector repertoires plays a major role in determining host range and virulence capabilities (Laflamme et al., 2020).

T3SE proteins have a range of virulence functions in plants, including suppressing immune responses, altering hormone signaling, disrupting cellular processes, and creating favorable conditions for bacterial growth (Khan et al., 2018). Some effectors, like HopZ1a, demonstrate remarkable functional diversity, targeting multiple unrelated plant proteins to suppress immunity, alter phytohormone signaling, and disrupt microtubule integrity (Jiang et al., 2013; Lee et al., 2012; Rufián et al., 2021). However, plants have evolved immune receptors capable of recognizing many effectors, triggering strong defense responses called effector-triggered immunity (ETI) (Jones and Dangl, 2006). This creates an evolutionary arms race, with bacteria evolving new or modified effectors to evade detection, and plants evolving new receptors.

Interestingly, recent research has found that a relatively small number of plant immune receptors are capable of recognizing effectors from a wide range of *P. syringae* strains, providing broad-spectrum resistance. For example, in *Arabidopsis thaliana*, the ZAR1 and CAR1 receptors can detect effectors from 95% of *P. syringae* strains (Laflamme et al., 2020). The ZAR1 receptor in particular shows a remarkably broad recognition profile, capable of detecting at least six unrelated T3SE families through association with multiple kinase proteins (Martel et al., 2020).

The evolution and diversity of *P. syringae* effector repertoires is shaped by several key processes (Dillon et al., 2019). Horizontal gene transfer allows effectors to move between strains, potentially expanding host range. Gene loss or pseudogenization can occur when effectors are recognized by plant defenses. Mutational changes in effector sequences may alter their function or allow evasion of host recognition (Dillon et al., 2019). Some effectors show functional redundancy, allowing loss of one effector to be compensated for by others (Kvitko et al., 2009). Additionally, some effectors can suppress the immune responses triggered by other effectors, a phenomenon known as meta effector interactions (Wei et al., 2018).

The importance of environmental factors in *P. syringae* infections should not be overlooked. Temperature, humidity, and leaf wetness play crucial roles in disease development. For example, optimal conditions for infection include temperatures around 15–25°C and periods of high humidity or leaf wetness (Xin et al., 2018). These environmental factors influence both bacterial growth and the plant’s immune responses. *P. syringae* is not limited to agricultural settings, it has also been isolated from various environmental sources including aquatic habitats, rain, and wild plants (Morris et al., 2013). This environmental ubiquity may contribute to the emergence of new plant diseases as strains adapt to new hosts or environmental conditions change.

Despite the existence of pathogenic strains, the vast majority of isolated and characterized *Pseudomonas* strains are beneficial or benign (Palleroni, 2015; Peix et al., 2009). Only a fraction of *Pseudomonas* strains, out of the numerous species identified, are pathogenic (Palleroni, 2015), highlighting the potential for screening diverse strains to identify those suitable for biocontrol applications. Below, we examine the diverse mechanisms through which *Pseudomonas* spp. contribute to the suppression of cereal crop diseases. Additionally, we explore the practical applications of these strains in agriculture and discuss the factors influencing their competitiveness and efficacy as biocontrol agents.

## 4 Control of plant diseases in cereal crops

*Pseudomonas* spp. offer significant potential for managing plant diseases in cereal crops. For instance, when rice seeds undergo treatment with *Pseudomonas fluorescens* PF1 before sowing, at the time of sowing, and again at 30 days after sowing, the resulting seedlings display enhanced resistance to *Xanthomonas oryzae* pv. *pryzae* (Vidhyasekaran et al., 2001). This treatment led to a significant decrease in disease incidence from 6.8 to 1.2%. In both greenhouse and field conditions, *P. fluorescens* strains PF1 and FP7 exhibit the ability to inhibit the mycelial growth of the sheath blight fungus *Rhizoctonia solani*. This inhibition contributes to improved seedling vigor and increased yield in rice plants (Nandakumar et al., 2001). Moreover, the treatment of rice cv. IR50 with the same *Pseudomonas* strains induces systematic resistance against *R. solani*, accompanied by an increase in chitinase and peroxidase activity (Nandakumar et al., 2001).

Field trials conducted with rice across different seasons evaluate the efficacy of *P. fluorescens* strain Pf-1 in controlling *Hirschmanniella gracili*s, root endoparasitic nematodes that are common in aquatic environments. The seed treatment with this biocontrol strain leads to a high level of bacterial colonization, suppression of nematodes, and a 13% increase in rice yield (Ramakrishnan et al., 1998).

In the case of wheat (*Triticum aestivum L*.), studies have shown that two *Pseudomonas* strains, GRP3 and PRS9, play a role in promoting wheat growth in terms of root-shoot length and plant weight (Sharma et al., 2011). *P. fluorescens* strain PSR21, when applied as a wheat seed treatment and later as a foliar spray during the spring, results in a significant decrease in the average degree of culm damage (where the culm is the above-ground stem of a grass or sedge) (Kita et al., 2004). Further research has highlighted the efficacy of *P. fluorescens* in reducing disease incidence, such as that caused by the fungal pathogen *Helminthosporium sativum* in wheat. Interestingly, following treatment, the bacterial population tends to decrease toward the root tip in wheat plants (Srivastava et al., 1999; Wang et al., 1999). Moreover, a recent study has investigated the effectiveness of eight *Pseudomonas* strains as biocontrol agents (Clough et al., 2022). Among these, *Pseudomonas protegens* CHA0 was identified as the most potent strain, significantly inhibiting the growth of *Ralstonia solanacearum*, a pathogen causing bacterial wilt in plants. This inhibitory activity was linked to the production of key secondary metabolites, including orfamides, pyoluteorin, and 2,4-diacetylphloroglucinol (DAPG) (Clough et al., 2022).

*Pseudomonas* spp. hold immense potential for combating plant diseases in cereal crops, offering multifaceted strategies for disease management. From rice to wheat, the application of various *Pseudomonas* strains has shown remarkable results, including reduced disease incidence, enhanced seedling vigor, and increased crop yield. These findings underscore the importance of developing microbial-based solutions in agriculture to mitigate the impact of plant pathogens on crop production.

## 5 Direct inhibition of phytopathogens

### 5.1 Antimicrobial and antifungal compounds

In the pursuit of novel biocontrol agents, *Pseudomonas* spp. have garnered considerable attention for their potential to inhibit some of the most damaging cereal crop pathogens. Efforts to isolate *Pseudomonas* strains from diverse environments have revealed promising candidates capable of combating phytopathogens (Chaudhary et al., 2021; Lv et al., 2023). Several studies have identified *Pseudomonas* species with the ability to inhibit numerous cereal crop pathogens, including *Magnaporthe oryzae* (causing rice blast), *Gaeumannomyces graminis* var. *tritici* (a fungus responsible for wheat take-all disease), *Fusarium* spp. (associated with head blight, root rot, wilt, and grain contamination), *Pyrenophora tritici-repentis* (a fungal pathogen that is the cause of tan spot disease), and *Rhizoctania solani* (a wide-host-range soil-borne pathogen) (Garbeva et al., 2004; Karthikeyan and Gnanamanickam, 2008; Lemanceau and Alabouvette, 1993; Rangarajan et al., 2003; Thomashow and Weller, 1988) (Table 1). These findings mark the initial steps in the quest to identify effective biocontrol agents, demonstrating the ability of isolates to confer plant protection *in vivo*, through greenhouse experiments and field trials.

**Table 1:** Effects of *Pseudomonas* spp. against plant bacteria and fungi pathogens.

*Pseudomonas* species	Target organism	Effect	Molecules/molecular systems	References
*P. fluorescens* PF1	*X. oryzae* pv. *pryzae*, *R. solani*	Growth inhibition	–	Nandakumar et al. (2001) and Vidhyasekaran et al. (2001)
*P. fluorescens* FP7	*R. solani*	Growth inhibition	Pyrrol o[1,2-a] pyrazine- 1,4-dione, hexahydro −3-(2-meth ylpropyl)- and Pyrrol o[1,2-a] pyrazine- 1,4-dione, hexahy dro-3-(pro pylmethyl)	Marrez et al. (2019) and Nandakumar et al. (2001)
*P. fluorescens* strain PSR21	Unspecified plant pathogen	Decrease in culm damage	–	Kita et al. (2004)
*P. fluorescens* Pf2-79r	Tilletia laevis	Growth inhibition	–	McManus et al. (1993)
*P. fluorescens* Pf-52	*Setaria italica*, *Magnaporthe oryzae*	Growth inhibition	Bacteriocins	Karthikeyan and Gnanamanickam (2008)
*P. fluorescens* 2–79	*Gaeumannomyces graminis* var. *tritici*	Growth inhibition	Phenazines	Thomashow and Weller (1988)
*P. fluorescens* Pf-5	*Pyrenophora tritici-repentis*	Growth inhibition	Pyrrolnitrin, c-Acetyl phloroglucinols	Loper and Gross (2007)
*P. fluorescens* DR54	*R. solani*, *Pythium ultimum*	Growth inhibition	Viscosinamide	Loper and Gross (2007)
*P. fluorescens* 96.578	*R. solani*, *Pythium ultimum*	Growth inhibition	Tensin	Nielsen et al. (2000)
*P. fluorescens* SS101	*Phytophthora infestans*	Growth inhibition	Massetolide	Tran et al. (2007)
*P. putida* A12	*Fusarium* spp.	Mitigated Fusarium wilts	–	Lemanceau and Alabouvette (1993) and Van Peer et al. (1990)
*P. fluorescens* Mst 8.2	*R. solani*	Growth inhibition	–	Gull and Hafeez (2012)
*P. fluorescens* Lp1	*Aspergillus flavus*, *Curvularia* spp., *Fusarium* spp.	Growth inhibition	–	Kanimozhi and Perinbam (2011)
*Pseudomonas* sp. PICF6	*Verticillium* spp., *Fusarium* spp., *Rhizoctonia* spp.	Growth inhibition	Volatile organic compounds	Montes-Osuna et al. (2022)
*P. simiae* PICF7	*Verticillium* spp., *Fusarium* spp., *Rhizoctonia* spp.	Growth inhibition	Volatile organic compounds	Montes-Osuna et al. (2022)
*P. putida* KT2440	*Xanthomonas campestris*	Growth inhibition	T6SS	Bernal et al. (2017)
*P. mosselii* BS011	*Magnaporthe oryzae*	Growth inhibition	Xantholysin	Wu et al. (2018)

Several greenhouse and growth chamber studies have explored the use of bioactive compounds purified from cultures of *Pseudomonas* species (Table 1) (Ligon et al., 2000; Wu et al., 2018). For instance, metabolites from *Pseudomonas mosselii* BS011 exhibited broad-spectrum inhibition against phytopathogenic fungi including *M. oryzae* (Wu et al., 2018). Importantly, *Pseudomonas* strains have demonstrated equal inhibition of pathogens compared to standard chemical fungicides (Ligon et al., 2000). For example, culture filtrates of *P. fluorescens* strain BL915 exhibited equal efficacy against *R. solani* compared to quintozene, a conventional chemical fungicide (Ligon et al., 2000).

*Pseudomonas* spp. Metabolites multiple compounds which contribute to their antagonistic properties. For example, the antagonistic properties of *P. protegens* FD6 were examined against pathogenic fungi such as *Botrytis cinerea* and *Monilinia fructicola* (Zhang et al., 2020). Genomic analysis identified 12 gene clusters responsible for the production of key secondary metabolites, including 2,4-diacetylphloroglucinol (2,4-DAPG), pyoluteorin (PLT), and pyrrolnitrin (PRN), all essential for its antifungal activities. Mutant analysis revealed that the pltD mutant, lacking PLT production, exhibited only 30% inhibition of grey mold disease, while the phlC mutant, deficient in 2,4-DAPG production but with a marked increase in PLT, demonstrated 65% inhibition. In contrast, the wild-type FD6 strain showed complete suppression of the pathogen, with no visible disease lesions on tomato fruits after 5 days of treatment. These findings underscore the critical roles of PLT and 2,4-DAPG, with PLT being particularly crucial for the high-level inhibition of fungal pathogens. The inverse relationship between the production of these two metabolites highlights the complexity of the biocontrol mechanisms in *P. protegens* FD6 (Zhang et al., 2020).

For wheat take-all disease, *Pseudomonas* spp. have been used as potential alternatives to conventional chemical treatments. *Pseudomonas* species offer resilience under unfavorable conditions and exhibit promising colonization abilities with cereal crop roots (Al Zadjali et al., 2023; Capper and Higgins, 1993; Dowling and O'Gara, 1994; Thomashow and Weller, 1988; Xu et al., 2021). Thus, the exploration of *Pseudomonas*-based biocontrol agents holds significant promise in augmenting sustainable agricultural practices, heralding a new era in crop protection strategies.

### 5.2 Siderophores

Siderophores constitute a group of secondary metabolites synthesized by various bacteria, fungi, yeast, and specific plants in response to iron scarcity (Dwivedi and Johri, 2003). These low molecular weight molecules, typically ranging from 500–1,500 Daltons, exhibit a strong binding affinity for iron (III) (Hider and Kong, 2010). The term “siderophore” is derived from the Greek, meaning “iron carrier” (Neilands, 1995); the first siderophore was isolated during 1949–1952 (Hider and Kong, 2010). The first crystalline form of siderophore was isolated by Neilands (Neilands, 1952), sparking research into these iron-chelating compounds (Chincholkar et al., 2000). Kloepper et al. (1980) provided initial evidence of siderophores from plant growth-promoting bacteria (PGPB) acting as biocontrol agents. The ability of an organism to produce siderophores is closely related to cyanide production, and their absence can impact the biocontrol activity of the microbes and their ability to restrict iron access to target pathogens (Ho et al., 2021; Jha et al., 2011). Iron plays an important role in the life of nearly all living organisms (Krewulak and Vogel, 2008).

Plants rely heavily on iron for various vital processes such as photosynthesis, oxygen metabolism, DNA and RNA synthesis, and more (Aznar et al., 2015; Rout and Sahoo, 2015). However, in many natural environments with aerobic conditions and neutral pH levels, iron exists mainly in its oxidized ferric (Fe^3+^) state, which is largely insoluble and limits its availability to plants (Colombo et al., 2014). The siderophores act as high-affinity chelating agents to solubilize ferric ions and transport them to the plant or bacterial cell where it is converted to Fe^2+^ (Kramer et al., 2020). Moreover, restricting the supply of iron to pathogens acts as a strategy to hinder their growth, owing to the insolubility of ferric iron forms (Kramer et al., 2020; Lau et al., 2016).

Siderophores can take up the iron available in the surrounding environment, and subsequently, iron is acquired by the organisms possessing receptors for that siderophore-iron complex, making it unavailable to their competitors (Hakim et al., 2021). Besides being used as iron carriers, bacterial siderophore-iron complexes can also be utilized by certain plants aiding in their better growth (Pathak et al., 2017). Among the Gram-negative bacteria where over 90% of the bacterial population produces siderophores, the most dominant genera are *Enterobacter* and *Pseudomonas* (Tian et al., 2009). Among *Pseudomonas* species, pyoverdine is the predominant siderophore observed (Cornelis and Matthijs, 2002; Ghssein and Ezzeddine, 2022).

Siderophores primarily contribute to the survival and fitness of microbial populations, directly and indirectly enhancing the activity of biocontrol species (Gull and Hafeez, 2012; Kanimozhi and Perinbam, 2011). For example, siderophores from *P. fluorescens* strain Mst 8.2 were shown to be a potent inhibitor against *R. solani*, with 70% disease reduction in wheat (Gull and Hafeez, 2012). Similarly, siderophores from *P. fluorescens* Lp1 have been used to inhibit plant fungal pathogens such as *Aspergillus flavus*, *Curvularia* spp., and *Fusarium* spp. (Kanimozhi and Perinbam, 2011).

### 5.3 Volatile organic compounds

*Pseudomonas* species are known to produce a wide array of volatile organic compounds (VOCs) with various functions and applications in agricultural settings (Hernández-León et al., 2015). These VOCs are small molecules characterized by low molecular weights and high vapor pressures, allowing them to diffuse effectively through soil pores and influence microbial communities and plant health (Wheatley, 2002).

*Pseudomonas* strains can generate complex mixtures of VOCs, although the full extent of their functional diversity is still being elucidated (Schmidt et al., 2015). Some VOCs produced by *Pseudomonas* spp. exhibit antimicrobial properties against phytopathogens, making them potential candidates for biocontrol applications. For instance, studies have identified *Pseudomonas*-derived VOCs with antifungal activity against a range of plant pathogens, including *Verticillium* spp., *Fusarium* spp., and *Rhizoctonia* spp. (Cordero et al., 2014; Elkahoui et al., 2015; Montes-Osuna et al., 2022). *Pseudomonas* sp. PICF6 produces 20 VOCs, that include compounds with reported antifungal (e.g., 1-undecene, (methyldisulfanyl) methane and 1-decene) or plant growth promoting (e.g., tridecane, 1-decene) activities (Montes-Osuna et al., 2022). These findings suggest that *Pseudomonas*-derived VOCs could serve as biofumigants to mitigate the proliferation of pathogenic species and promote plant health.

Further studies have investigated the antifungal activity of VOCs produced by *P. fluorescens* ZX against postharvest fungal pathogens (Wang et al., 2021; Yue et al., 2023). The research demonstrates that *P. fluorescens* ZX VOCs effectively inhibit the growth of both *Penicillium italicum* and *Botrytis cinerea*, causative agents of blue mold in citrus and gray mold in various fruits, respectively (Wang et al., 2021; Yue et al., 2023). The VOCs significantly suppressed mycelial growth, conidial germination, and sporulation of these pathogens *in vitro* and reduced disease incidence and lesion size on infected fruits *in vivo*. Mechanistically, the VOCs primarily act by damaging the pathogens’ cell membrane integrity and permeability, leading to cellular content leakage, decreased ergosterol biosynthesis, and increased malondialdehyde content (Wang et al., 2021; Yue et al., 2023). Additionally, the VOCs interfere with pathogen respiration by inhibiting key enzymes such as ATPase, malate dehydrogenase, and succinate dehydrogenase, causing energy metabolism disruption and reactive oxygen species accumulation. Transcriptomic analysis revealed significant changes in gene expression related to membrane components and amino acid metabolism pathways in treated pathogens (Wang et al., 2021; Yue et al., 2023).

However, to fully harness the potential of *Pseudomonas* VOCs for practical applications, further investigations are necessary to fully characterize their production within the plant root system and assess their efficacy under natural environmental conditions. By exploring the roles of *Pseudomonas*-derived VOCs in plant-microbe interactions, researchers can unlock valuable insights into sustainable strategies for crop protection and soil management.

### 5.4 Anthelmintic compounds

*Pseudomonas* spp., in addition to their antimicrobial activities, have also demonstrated the capability to produce potent anthelmintic compounds, they can destroy parasitic worms and nematodes (Devaraj et al., 2019; Lee C. H. et al., 2000). While not as extensively studied in *Pseudomonas* as in *Streptomyces*, the potential for nematode control exists within this bacterial genus (Table 2) (Devaraj et al., 2019; Lee C. H. et al., 2000; Rahanandeh et al., 2024; Spiegel et al., 1991; Wang et al., 2024a; Wang et al., 2024b).

**Table 2:** Effects of *Pseudomonas* spp. on nematodes and insects.

*Pseudomonas* species	Target organism	Effect	Molecules/molecular systems	References
*P. fluorescens* Pf-1	*Hirschmanniella gracilis*	Nematode suppression	–	Ramakrishnan et al. (1998)
*P. fluorescens*	*Meloidogyne javanica*	Reduced nematode infection, egg mass destruction, decreased egg hatching	–	Norabadi et al. (2014)
*P. chlororaphis* PcR3-3	*Plagiodera versicolora*	High insecticidal activity	Chitinase C, phospholipase C	Wang et al. (2024a)
*P. fluorescens*, *P. aeruginosa*	*Pratylenchus loosi*	Strong nematicidal activity	Metalloproteases, serine proteases	Rahanandeh et al. (2024)
*P. simiae* MB751	*Meloidogyne incognita*	Nematicidal activity	Cyclo(L-Pro-L-Leu)	Sun et al. (2021)

Root-knot nematodes (*Meloidogyne* spp.) are among the most economically damaging plant-parasitic nematodes worldwide, causing significant yield losses in many crops (Sun et al., 2021). These microscopic roundworms infect plant roots, forming characteristic galls or “knots” that disrupt water and nutrient uptake. *Meloidogyne incognita* is one of the most widespread and studied species, with a broad host range and short generation time that makes it particularly difficult to control.

One study investigated the potential of a strain of *P. fluorescens* as a biocontrol agent against *Meloidogyne javanica*, another important root-knot nematode species (Norabadi et al., 2014). In greenhouse and laboratory experiments, *P. fluorescens* application significantly reduced nematode infection compared to the control group. The bacteria destroyed the nematode egg mass matrix and decreased egg-hatching levels. Additionally, *P. fluorescens* inoculation led to increased activities of peroxidase (POX) and phenylalanine ammonia lyase (PAL), key enzymes associated with plant defense mechanisms (Norabadi et al., 2014). These observations suggest that *P. fluorescens* effectively suppress nematode populations by disrupting egg structures and inducing systemic resistance in plants.

Recent research has expanded our understanding of *Pseudomonas* spp. potential for control of both nematodes and insect pests (Wang et al., 2024a; Wang et al., 2024b). For example, *Pseudomonas chlororaphis* PcR3-3, isolated from willow roots, exhibited high insecticidal activity against the coleopteran pest *Plagiodera versicolora* (Wang et al., 2024a). This leaf beetle is a significant pest of willow and poplar trees. While this study primarily focused on insecticidal activity, it highlights the diverse capabilities of *Pseudomonas* strains against different invertebrates. The genome of *P. chlororaphis* PcR3-3 was found to contain several genes potentially involved in insect pathogenicity, including those encoding chitinase C and phospholipase C (Wang et al., 2024b). These enzymes have been previously associated with the disruption of insect gut structures and could potentially play a role in nematode control as well.

Other studies have demonstrated the nematicidal potential of *Pseudomonas* spp. against the root lesion nematode *Pratylenchus loosi*, a significant pest of tea plants (Rahanandeh et al., 2024). It was found that *P. fluorescens* and *Pseudomonas aeruginosa* strains, which were isolated from tea rhizosphere, produced proteases with strong nematicidal activity. These proteases were capable of completely degrading *Pratylenchus loosi* nematodes within 8 h of exposure (Rahanandeh et al., 2024). Characterization of the proteases revealed them to be metalloproteases and serine proteases, classes of enzymes known to be involved in the degradation of nematode cuticles.

Although the research exploring *Pseudomonas* spp. as a source of anthelmintic compounds is relatively limited in Pseudomonas compared to other prokaryotes, these studies have indicated the potential of certain *Pseudomonas* strains to control populations of both plant-parasitic nematodes and insect pests. Given the diverse array of natural products synthesized by *Pseudomonas* strains and their interactions within soil ecosystems, it is likely that additional anthelmintic compounds may be discovered among *Pseudomonas* spp. Metabolites (Devaraj et al., 2019). This highlights the potential of *Pseudomonas* species as versatile biocontrol agents capable of addressing multiple agricultural pest problems.

### 5.5 Type VI secretion system

The Type VI Secretion System (T6SS) has emerged as a pivotal weapon in bacterial warfare, allowing bacteria to deliver toxic effectors directly into neighboring cells (Mougous et al., 2006; Pukatzki et al., 2006). T6SSs are present in more than 25% of gram-negative bacteria (Ho et al., 2014). The T6SS is a contact-dependent secretion system that plays a crucial role in interbacterial competition (Mougous et al., 2006; Pukatzki et al., 2006). It provides a selective advantage to producer strains by annihilating competitors either in an indiscriminate manner or in response to danger signals (Basler et al., 2013; Durán et al., 2021; Ho et al., 2014; Hood et al., 2010). The T6SS was originally described in *Vibrio cholerae* and *P. aeruginosa* as a proteinaceous nanomachine that translocates specific proteins directly into target cells (Mougous et al., 2006; Pukatzki et al., 2006). It was later observed and analyzed in many other bacterial pathogens (Burtnick et al., 2011; De Pace et al., 2010; Murdoch et al., 2011; Suarez et al., 2008). However, the analytical description of T6SS in non-pathogenic bacteria is underrepresented in the scientific literature (Bernal et al., 2017; Durán et al., 2021; Marchi et al., 2013).

The killing activity conferred by antibacterial T6SSs can be extremely potent during *in vitro* co-culture experiments, with T6SS-wielding cells often able to virtually eliminate similar numbers of susceptible competitor cells within a few hours (Basler et al., 2013; Durán et al., 2021; Ho et al., 2014). Antibacterial T6SSs are frequently found in both pathogenic and symbiotic or beneficial plant-associated bacteria (Bernal et al., 2018; Durán et al., 2021). This suggests that antibacterial T6SSs may be involved in establishing and protecting beneficial plant-associated communities from invasion of these communities by pathogens. For example, the T6SS-dependent antibacterial activity in the rhizosphere bacterium *P. putida* KT2440 contributes to reducing colonization and necrosis induced by the phytopathogen *Xanthomonas campestris* when both are co-infiltrated into *Nicotiana benthamiana* leaves (Bernal et al., 2017).

Further *in planta* studies show that T6SS in *P. fluorescens* MFE01 play crucial role in protecting potato tubers from *Pectobacterium atrosepticum* infection (Bourigault et al., 2023). Fluorescence microscopy revealed that MFE01 exhibits an aggressive T6SS behavior with continuous and intense firing activity, causing rounding and lysis of target *P. atrosepticum* cells. The study identified a putative T6SS-secreted amidase effector, Tae3Pf, as a major contributor to MFE01’s antibacterial activity. This effector was found to be toxic when produced in the periplasm of *Escherichia coli*, with its toxicity neutralized by the inner membrane immunity protein Tai3Pf. While Tae3Pf plays a significant role, the research suggests that other T6SS effectors likely contribute to MFE01’s overall antibacterial activity (Bourigault et al., 2023). These findings highlight the importance of the T6SS in *P. fluorescens* MFE01’s biocontrol capabilities, offering new perspectives on bacterial antagonism in the context of plant protection and potentially leading to the development of more effective biocontrol strategies in agriculture.

## 6 Indirect inhibition of phytopathogens

In addition to direct inhibition via the production of antagonistic compounds, *Pseudomonas* spp. demonstrate a remarkable capacity to indirectly inhibit plant pathogens (Haney et al., 2018; Leeman et al., 1995). Competitive exclusion is one of the primary mechanisms through which *Pseudomonas* strains exert their inhibitory effects (Archetti et al., 2011; Buddrus-Schiemann et al., 2010; Rainey, 1999). By occupying niche spaces and consuming available resources, these strains effectively thwart the colonization efforts of pathogens, preventing them from establishing robust populations and causing harm to plants (Archetti et al., 2011; Buddrus-Schiemann et al., 2010; Rainey, 1999). This strategy, however, does not operate in isolation; rather, it complements direct antagonism, wherein *Pseudomonas* spp. secretes antimicrobial compounds that further hinder pathogen growth and proliferation (Haney et al., 2018; Leeman et al., 1995). These antimicrobials may arise as byproducts of interference competition over resources provided via plant root exudates or organic matter in the soil, illustrating the multifaceted nature of *Pseudomonas*-mediated inhibition.

*Pseudomonas* spp. possess the ability to activate host resistance pathways, thereby inducing systemic resistance (ISR) in plants (Table 3) (Chen et al., 1999; Elsharkawy et al., 2022; Haney et al., 2018; Hoffland et al., 1996; Leeman et al., 1995; Meziane et al., 2005; Nandakumar et al., 2001). ISR is a state of enhanced defensive capacity developed by a plant reacting to specific biotic or chemical stimuli. *Pseudomonas* spp. mediate ISR through phytohormone defense signaling pathways such as jasmonic acid/ethylene (JA/ET) or salicylic acid (SA) (Chen et al., 1999; Pangesti et al., 2016). For instance, a study investigated the efficacy of *Pseudomonas corrugata* strain 13 and *Pseudomonas aureofaciens* strain 63–28 in inducing systemic resistance against *Pythium aphanidermatum* in cucumber roots (Chen et al., 1999). Both bacterial strains were found to produce SA *in vivo* and induced cucumber roots to accumulate endogenous SA within a day of inoculation (Chen et al., 1999). Notably, plants treated with a *Pseudomonas* strain showed significantly elevated SA levels compared to the control, persisting from 1 to 5 days post-bacterization (Chen et al., 1999). Interestingly, SA inhibited the mycelial growth of the pathogen *P. aphanidermatum* at higher SA concentrations (Chen et al., 1999).

**Table 3:** *Pseudomonas* species inducing systemic resistance (ISR) in plants.

*Pseudomonas* species	Host plant	Pathogen/Pest controlled	References
*P. protegens* CHA0	Tomato	*Meloidogyne javanica*	Siddiqui and Shaukat (2002)
*P. fluorescens* WCS417r	Arabidopsis, Tomato	Various pathogens	Ab Rahman et al. (2018)
*P. putida* WCS358	Arabidopsis	Various pathogens	Meziane et al. (2005)
*P. fluorescens* Pf1	Rice	*R. solani*	Nandakumar et al. (2001)
*P. fluorescens* FP7	Rice	*R. solani*	Nandakumar et al. (2001)
*P. fluorescens* 63–28	Cucumber	*P. aphanidermatum*	Chen et al. (1999)
*P. fluorescens* SS101	Tomato	*Phytophthora infestans*	Tran et al. (2007)
*P. simiae* MB751	Tomato	*Meloidogyne incognita*	Sun et al. (2021)

In response to infections by various pathogens, such as fungi, bacteria, or nematodes, plants often produce stress ethylene (Abeles et al., 1992; Iqbal et al., 2017). The symptoms observed in an infected plant largely stem from the stress caused by the pathogen (Iqbal et al., 2017; Van Loon, 1984). A significant portion of the damage to infected plants results from the plant’s response to increased levels of stress ethylene (Stearns and Glick, 2003). Exogenous ethylene can exacerbate pathogen infections, while chemical inhibitors of ethylene synthesis can reduce their severity (Stearns and Glick, 2003; Yang et al., 2017). Additionally, pretreating plants with ACC deaminase-containing plant growth-promoting bacteria can provide significant protection against ethylene-induced damage from pathogen infections (Glick et al., 1995; Hao et al., 2011; Toklikishvili et al., 2010; Wang et al., 2000). Some bacteria that promote plant growth encode the enzyme ACC deaminase, which breaks down ACC exudates from plants, the direct precursor of ethylene production, and lowers the amount of ethylene that plants produce in response to different stressors (Glick et al., 1995; Hao et al., 2011). Many other studies showed that the application of ACC-deaminase-producing plant-growth-promoting bacteria was found to inhibit the growth of phytopathogens in crops (Toklikishvili et al., 2010; Wang et al., 2000).

The ability of *Pseudomonas* spp. to induce plant disease resistance holds significant promise for its application as a biocontrol agent (Haney et al., 2018; Leeman et al., 1995). Strains that exhibit such abilities may prove highly effective at protecting their plant hosts against pathogenic infections *in situ*, even if they demonstrate poor bioactivity against phytopathogens *in vitro*. This underscores the importance of screening for biocontrol strains based not only on their performance in traditional *in vitro* bioactivity assays but also on their ability to elicit host defenses and confer protection to host plant species *in vivo*. As research in this field progresses, better proxies, and methodologies for evaluating the efficacy of *Pseudomonas* strains as biocontrol agents should be developed, considering the intricate interplay between bacterial-induced plant defenses and pathogen susceptibility.

## 7 The potential use of *Pseudomonas* species as effective biocontrol agents

Many studies demonstrate promise in laboratory settings but exhibit inconsistent efficacy in agricultural settings (Arora et al., 2008; Garbeva et al., 2004; Lee H. S. et al., 2000; Rini and Sulochana, 2007). The inconsistent results of biocontrol treatments show we need to better understand what affects soil and root microbes. Abiotic factors such as soil type, climate, and farming practices, along with biotic factors like host crop species and root exude profiles, profoundly influence microbial assemblages and biocontrol success (Babin et al., 2019; Classen et al., 2015; Edwards et al., 2015; Glick and Gamalero, 2021; Rousk et al., 2010). Efforts such as the Microbiome Stress project aim to understand bacterial community responses to environmental stressors, aiding in the development of robust biocontrol strategies resilient to changing environmental conditions and advancing sustainable agriculture practices (Rocca et al., 2019).

### 7.1 Abiotic factors influencing biocontrol efficacy

Numerous studies have focused on enhancing the effectiveness and consistency of *Pseudomonas* spp. biocontrol agents through various agricultural practices and interventions (Zhang et al., 2015). This includes the identification of several minerals and carbon sources that have a differential influence on the production of the antibiotics 2,4-diacetylphloroglucinol, pyoluteorin and pyrrolnitrin and the salicylic acid and pyochelin by disease-suppressive strains of *P. fluorescens* (Duffy and Défago, 1999). For example, the addition of glycerol, zinc and ammonium molybdate to *P. protegens* CHA0 has shown promise in increasing its biocontrol efficacy against *Meloidogyne javanica* (Hamid et al., 2003; Siddiqui and Shaukat, 2002). Additionally, adding zinc to soil containing *P. fluorescens* PfAs1 has enhanced the expression of genes resistant to bacterial leaf blight in rice, while also significantly reducing bacterial leaf blight in rice caused by *X. oryzae* pv. *oryzae* (Sharma et al., 2020). Similarly, supplementing *P. fluorescens* NK2 with the zinc oxide nanoparticle (ZnO-NP) enhances the inhibition efficacy against the pathogenic bacterium *Pseudomonas viridiflava* NK2 in cucumber (Al-Karablieh et al., 2022).

Beyond strain inoculation, various agricultural practices significantly impact the composition and establishment of species within the plant root microbiome. Practices like irrigation, tillage, and cropping methods can play crucial roles (Babin et al., 2019; Dennert et al., 2018; Mavrodi et al., 2018). Agro-chemicals such as pesticides and fertilizers also shape the plant root and soil microbiome, offering protection against crop diseases (Ding et al., 2013; Shen et al., 2019). For instance, ammonia fumigation effectively suppresses *Fusarium* wilt disease in bananas (*Musa acuminate Cavendish*) while inducing shifts in the soil microbial community, notably reducing *Fusarium* species abundance (Shen et al., 2019). Studies suggest that combining organic fertilizers with biocontrol strains enhances disease suppression further. Thus, the addition of biocontrol strains to organic fertilizers before application creates a more conducive soil environment, fostering nutrient availability, root colonization, and biocontrol effectiveness, a strategy known as bio-organic fertilizer application, widely recognized for its efficacy in disease suppression (Ding et al., 2013; Ketabchi et al., 2016; Watanabe et al., 1987).

Conversely, certain chemical additives and carbon compounds have been found to impede the biocontrol efficacy of *Pseudomonas* spp. For example, the addition of glucose can hinder the efficacy of *P. protegens* CHA0 against *Meloidogyne javanica* (Siddiqui and Shaukat, 2002). However, some *Pseudomonas* strains exhibit a high tolerance to commonly used fungicidal compounds, paving the way for synergistic approaches where chemical and biological pest control methods can be combined to enhance efficacy while reducing the overall pesticide dose (Anand et al., 2010; Kataria et al., 2002).

While these findings suggest the potential for optimizing farming practices to maximize disease suppression, comprehensive research in this area remains limited. The complexity of agricultural ecosystems and the variability in pathogen dynamics necessitates further investigation to identify the most effective strategies tailored to specific pathogens, climatic conditions, and soil characteristics. Nonetheless, exploring the interplay between *Pseudomonas* spp. biocontrol agents, agricultural practices, and chemical additives hold promise for sustainable disease management and crop protection.

### Optimizing biocontrol delivery systems involving *Pseudomonas*

**Figure 2:**
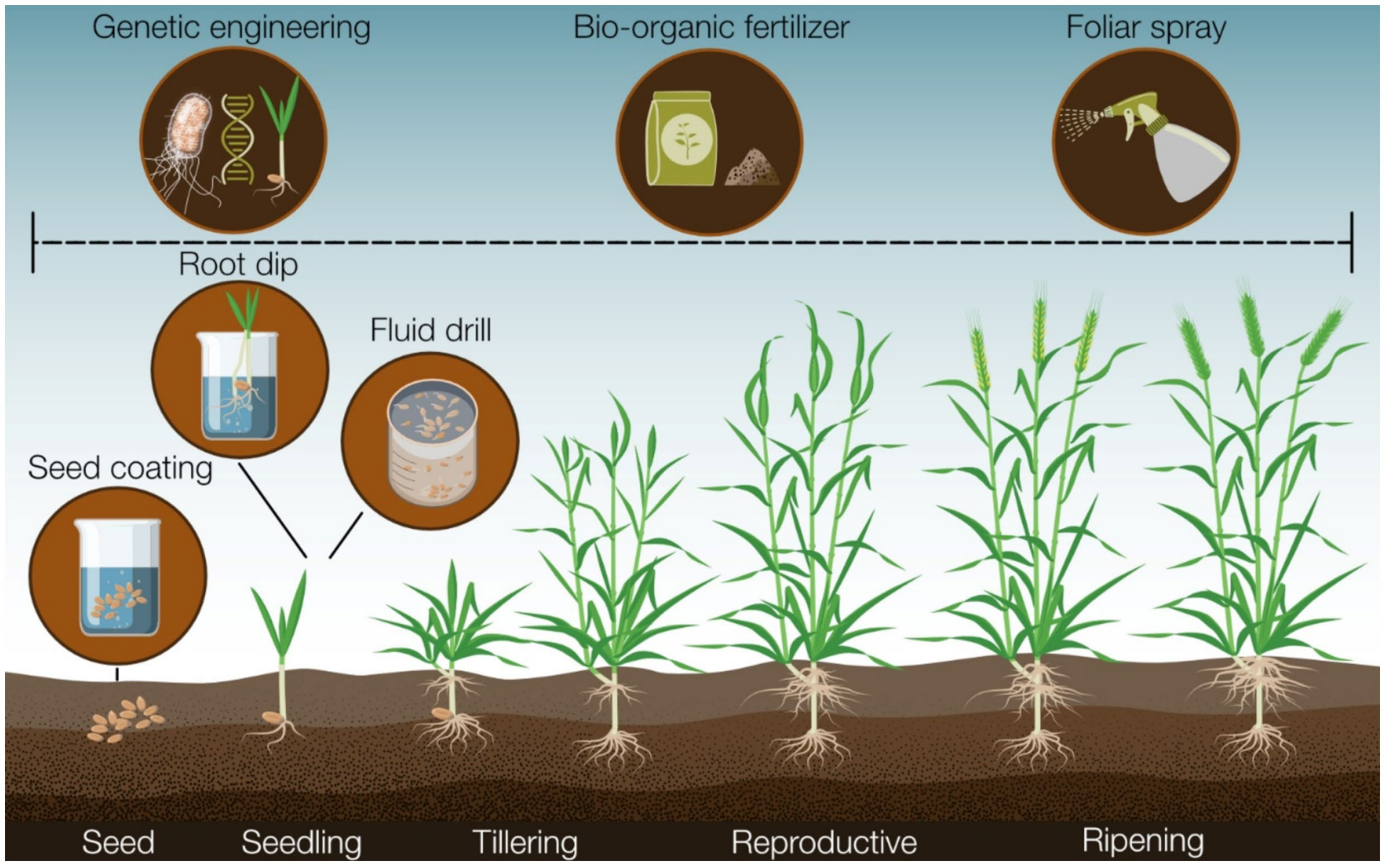
A summary of the major tools and methods available that could facilitate the application of biocontrol strains that can influence biocontrol efficacy in the field.

Numerous methods exist for delivering biocontrol strains to crops, each potentially influencing the consistency of biocontrol strategies (Jambhulkar et al., 2016; Preininger et al., 2018). Foliar spraying may seem appealing, especially in developed countries with available spraying equipment, but microbial suspensions can damage or clog machinery due to settling out of solution, and stresses from spraying apparatus (e.g., heat stress, shearing forces) can reduce biocontrol strain viability (Figure 2) (Preininger et al., 2018). Moreover, foliar spray that is suitable for microbial inoculants targeting foliar diseases (Jambhulkar et al., 2016) is often less effective for controlling root diseases like wheat take-all. Soil inoculation, another recommended method, is often employed if biocontrol strains are susceptible to desiccation (Jambhulkar et al., 2016). As discussed earlier, methods such as bio-organic fertilizer application (Ding et al., 2013; Ketabchi et al., 2016; Watanabe et al., 1987) can enhance biocontrol strain success. However, these strategies may increase the expense and complexity of applying disease-suppressive measures, while also potentially altering soil chemistry and microbiome composition with unknown or conflicting effects (Babin et al., 2019; Shen et al., 2019).

Direct inoculation of biocontrol strains onto plant roots offers a strategy to bypass challenges associated with soil-survivability as the strain bypasses exposure to an environmental medium before colonizing roots (Jambhulkar et al., 2016; Pill, 1991). Techniques like fluid drill inoculation and root transplant dip exemplify this approach, allowing biocontrol agents to colonize roots under controlled conditions (Jambhulkar et al., 2016; Pill, 1991). In root dip, seedling roots are immersed in a liquid cell suspension before field transfer, while in fluid drill methods, seeds pre-germinate within a gel containing the biocontrol strain (Jambhulkar et al., 2016; Pill, 1991). Root dip has demonstrated enhanced root colonization by *Pseudomonas* compared to soil inoculation in some cases (Pathak et al., 2004), effectively protecting crops against *R. solani*. Other studies showed that root dip was effective for *P. fluorescens* SBW25 to colonize the root of wheat (Guan et al., 2024). However, pre-germinating plants and manually inoculating roots are labor-intensive processes compared to purchasing pre-coated seeds (O’Callaghan, 2016). Similarly, fluid drill methods have shown increased root colonization by inoculated bacterial strains, in limited studies, indicating efficient disease suppression (Clarkson et al., 2002; Hardaker and Hardwick, 1978). Nevertheless, research exploring the ecological impact of fluid drill gel application remains sparse.

As mentioned, plants can also undergo colonization by coating the seed with a formulation of biocontrol strain spores or cells, employing various methods to adhere biocontrol strains to the seed surface. For instance, seeds may be submerged in a microbial suspension and subsequently dried before seed germination (known as bio-priming) (Callan et al., 1990), or a liquid cell suspension or adhesive may be utilized to coat the seed in bacterial cells (referred to as film coating) (O’Callaghan, 2016). Seed coating technologies effectively deliver biocontrol strains directly to the soil surrounding a germinating seed and the rhizosphere (Jambhulkar et al., 2016; O’Callaghan, 2016), with numerous instances demonstrating the efficacy of seed coating approaches in disease suppression in both field and laboratory settings (Ardakani et al., 2010; Callan et al., 1990; El-Mougy and Abdel-Kader, 2008; Ma et al., 2019; Prathuangwong et al., 2013). This includes several studies showcasing the effectiveness of seed coatings as delivery methods for *Pseudomonas* spp. biocontrol strains (Ardakani et al., 2010; Prathuangwong et al., 2013). In addition, scientists have recently reported developing a unique procedure to introduce endophytic bacteria into plant seeds so that it was no longer necessary to treat the seeds before planting (Glick and Gamalero, 2021).

While the focus of this review is on *Pseudomonas* as biocontrol agents, it is worth noting an emerging trend in the use of nanoparticle-based *Pseudomonas* biofertilizers. Although primarily applied for plant growth promotion rather than disease control, this innovative approach may have implications for biocontrol strategies. Nanoparticle-based biocontrol formulations have shown significant potential in overcoming delivery challenges (El-Ghamry et al., 2018). In these formulations, plant growth-promoting bacteria and nutrients are encapsulated within nanoscale polymers (nanoencapsulation) (Golbashy et al., 2017). This method enhances soil microbial activity, improves aeration, and supports natural fertilization (Itelima et al., 2018). For instance, selenium nanoparticles combined with *Pseudomonas* spp. have been used to improve crop nutrition and soil quality (Sonali et al., 2024) However, not all nanoparticles interact synergistically with *Pseudomonas* spp. (Dimkpa et al., 2012a; Dimkpa et al., 2012b). For example, zinc nanoparticles were found to enhance siderophore production more effectively than copper nanoparticles, which inhibited the expression of genes related to siderophore transport (Dimkpa et al., 2012a). These findings suggest that while nanoparticle-based biocontrol formulations hold significant promise in enhancing soil health and crop productivity, their interactions with specific biocontrol strains, such as *Pseudomonas* spp., are complex and not always synergistic.

The successful delivery of biocontrol strains remains a key challenge in modern agriculture, with methods ranging from foliar sprays to seed coatings and root inoculation. While each approach offers distinct advantages, the complexity of balancing cost, labor, and ecological impact must be carefully considered. Recent advancements, such as nanoparticle-based biocontrol formulations, hold great potential for improving efficacy and sustainability. However, the variability in interactions between nanoparticles and biocontrol strains like *Pseudomonas* spp. underscores the need for further research to optimize these technologies for widespread agricultural use.

### 7.3 Exploiting plant recruitment mechanisms to improve biocontrol agent outcomes

In addition to increasing the competitiveness of bacterial strains when applied to seeds and soil, leveraging the mechanisms by which plants selectively recruit microbial species from the soil could enhance the efficacy of biocontrol strains (Ryan et al., 2009; Zhang et al., 2015). Plants release approximately 20–40% of photosynthetically fixed carbon through their roots into the soil, comprising various compounds like ions, amino acids, sugars, phenolics, mucilage, polysaccharides, and proteins (Badri et al., 2013; Bais et al., 2006; el Zahar Haichar et al., 2016; Zhalnina et al., 2018). This root exudation triggers a surge in microbial abundance and activity in the soil surrounding the roots, known as the “rhizosphere effect,” as many microbes are attracted to the carbon-rich nutrients exuded from the roots (Bais et al., 2006); soil microbes come to plant rhizospheres to feed. Moreover, exudates potentially act as a filtering mechanism, enabling plants to selectively enrich for specific microbial species with certain metabolic capabilities (Bais et al., 2006). Experiments profiling the root exudates of *Arabidopsis thaliana* have shown that specific exudate compounds correlate with the abundances of specific bacterial taxa, indicating their role in microbial recruitment (Badri et al., 2013; Chaparro et al., 2013; Chaparro et al., 2014). Stable isotope probing experiments have further revealed that different microbial taxa metabolize plant root exudates based on differences in exudate composition (el Zahar Haichar et al., 2016; Haichar et al., 2008; Haichar et al., 2012). Additionally, root exudation can be influenced by abiotic and biotic factors, including responses to pathogens, which alter the microbial community composition (Jousset et al., 2011; Lanoue et al., 2010).

Beyond changes in their relative abundance, root exudates may alter the functionality of the root microbiome by modifying microbial gene expression (Chaparro et al., 2014). For instance, benzoxazinoid-related compounds, exuded by maize roots, correlate with an increased number of microbial transcripts related to beneficial *P. putida*, which resulted in significantly higher numbers of *P. putida* cells colonizing the root (Neal et al., 2012).

Understanding the relationships between root exudate composition, microbial community structure, and microbiome functionality offers promising avenues for enhancing crop productivity and health. Engineering plants to produce specific root exudates could improve the colonization potential and efficacy of beneficial species and biocontrol agents, such as *Pseudomonas* spp. (Ali and Glick, 2024). *Arabidopsis* mutant lines engineered with altered root exudation profiles have shown increased recruitment of beneficial rhizobacteria, suggesting similar approaches could be applied to cereal crops through breeding or genetic modification (Badri et al., 2009; Bressan et al., 2009; Huang et al., 2019). However, there remains a substantial knowledge gap regarding the compounds acting as signals and nutrients for bacteria of interest. While cues are well-defined for certain plant-microbe symbioses, many other systems lack a detailed understanding. Advanced tools like stable isotope probing, metabolomics, dual RNA sequencing, and imaging mass spectrometry are beginning to elucidate some of these interactions, paving the way for a deeper understanding of plant-microbe dynamics in the future (Berry et al., 2013; Camilios-Neto et al., 2014; el Zahar Haichar et al., 2016; Haichar et al., 2012; Zhalnina et al., 2018).

### 7.4 Engineered *Pseudomonas* for enhanced efficacy

It is generally assumed that suppression of plant pathogens by *Pseudomonas* spp. is based on two primary features: (1) production of natural products (direct inhibition), and (2) stimulation of ISR, which activates the plant defense system against harmful microbes and viruses. The expression of genes and natural products of *Pseudomonas* is influenced by growth and environmental conditions (Chin-A-Woeng et al., 2003; Hamid et al., 2003). These conditions can negatively affect the efficacy of *Pseudomonas* spp. as biocontrol strains (Nadeem et al., 2016). For instance, biocontrol *Pseudomonas* strains may perform well in one location or field season but not the next, owing to a wide range of biotic and abiotic factors in the soil environment that can adversely impact the *Pseudomonas* strain’s root colonization, expression of genes involved in biocontrol and/or activity of biocontrol metabolites (Babin et al., 2019; Classen et al., 2015; Edwards et al., 2015; Rousk et al., 2010).

Based on the increasing knowledge of the molecular mechanisms that underline natural product synthesis, the use of improved genetically modified biocontrol agents in agricultural seems feasible. However, the intentional release of genetically modified organisms (GMOs) requires extensive analysis of their potential ecological impact (Glick and Skof, 1986; Kolseth et al., 2015; Then et al., 2020). Moreover, the issue of whether GMOs could affect non-target organisms needs to be addressed. Laboratory experiments must be conducted before the release of GMOs into the natural environment.

Many studies have engineered *Pseudomonas* strains to enhance their biocontrol efficacy (Bakker et al., 2002; Barahona et al., 2011; Glandorf et al., 2001; Leeflang et al., 2002; Niemann et al., 1997; Shaukat and Siddiqui, 2003; Viebahn et al., 2003; Xiao-Jing et al., 2005). For instance, *P. putida* WCS358 was genetically modified to overproduce the antifungal compound 2,4-diacetylphloroglucinol (Phl) (Bakker et al., 2002; Glandorf et al., 2001; Leeflang et al., 2002), this genetically modified strain displays enhanced antifungal activity (Glandorf et al., 2001). Modified derivatives of strain *P. putida* WCS358 caused transient shifts in the composition of bacterial and fungal communities in the rhizosphere of wheat. However, they had no effect on soil metabolic activities (Bakker et al., 2002; Glandorf et al., 2001; Leeflang et al., 2002). In addition, a hypermotile mutant of *P. fluorescens* F113 was superior to the wild-type strain in colonizing the plant rhizosphere and controlling *Fusarium oxysporum* and *Phytophthora cactorum* pathogenesis (Barahona et al., 2011).

By harnessing the power of genetic modification, researchers may be able to tailor *Pseudomonas* strains to target specific pathogens while minimizing unintended ecological impacts (Barahona et al., 2011; Glandorf et al., 2001; Leeflang et al., 2002; Viebahn et al., 2003). These advancements pave the way for sustainable and environmentally friendly strategies to combat crop diseases, contributing to the overall resilience and productivity of agricultural systems. Continued research and innovation in this area holds promise for further enhancing the biocontrol potential of genetically modified *Pseudomonas* strains, ultimately benefiting farmers and ecosystems alike.

### 7.5 The biosafety of *Pseudomonas*-based biocontrol agents

Beyond these logistical hurdles, the safety concerns regarding clinical toxicity and environmental persistence pose additional obstacles to the commercialization of *Pseudomonas* spp. as biocontrol agents, necessitating extensive screening processes that impede progress (Fravel, 2005). Many candidate biocontrol agents are not well characterized and lack a thorough assessment of their non-target effects, leaving gaps in our understanding of their ecological impacts (Viaene et al., 2016; Winding et al., 2004).

An underlying concern with using antagonistic bacteria like *Pseudomonas* spp. as biocontrol agents is the diverse array of secondary metabolites they produce, which could potentially exert unintended non-target effects, including those detrimental to human health (Deising et al., 2017). While these metabolites effectively target pathogens, some may also pose risks to human cells by interacting with essential human metabolites, such as cholesterol (Zotchev, 2003). The actual production and concentration of antimicrobials by biocontrol agents in the soil remains inadequately explored (Deising et al., 2017; Winding et al., 2004). Some evidence suggests that the role of antimicrobials in inhibiting pathogens within the plant root niche might be minor compared to other traits like resource competition and immune system activation (Hennessy et al., 2017; Koch et al., 2018). The tight regulation of gene clusters encoding secondary metabolites raises the possibility that their activation depends on the direct interaction with specific pathogenic species, potentially minimizing non-target effects compared to the widespread use of purified antimicrobial molecules (Bertrand et al., 2014; Van der Meij et al., 2017). Nonetheless, deeper insights into the *in vivo* behavior and concentration of secondary metabolites produced by biocontrol agents are essential to develop strategies that mitigate non-target effects.

Notwithstanding several studies highlighting the influence of *Pseudomonas* spp. on individual plant symbionts, broader investigations into the impacts of these bacteria on the soil ecosystem and root microbiome are relatively scant (Balser et al., 2002; Glick and Gamalero, 2021; Khatoon et al., 2020; Lv et al., 2023). The introduction of biocontrol strains at low densities, such as through seed coatings, may have limited effects on the indigenous microbial community, especially in highly diverse soils (Koch et al., 2018). However, larger inoculations could modify existing soil communities, with some studies demonstrating short-term alterations in microbial composition following biocontrol strain application (Schmidt et al., 2014; Winding et al., 2004). Understanding the long-term implications of biocontrol strain application on pathogen populations, soil microbiome functionality, and environmental processes requires meticulously designed experiments, incorporating indicators like mycorrhizal abundance and functional gene expression related to plant-beneficial processes (Schmidt et al., 2014; Sisaphaithong et al., 2012; Winding et al., 2004). Rigorous controls are imperative to delineate the specific impacts of biocontrol *Pseudomonas* strains on soil microbiomes, paving the way for informed decision-making in sustainable agriculture (Winding et al., 2004).

Genetically modified biocontrol agents offer several potential advantages over conventional chemical pesticides. They can be engineered to have increased efficacy, broader host range, improved persistence in the environment, and enhanced production of antimicrobial compounds (Glandorf, 2019; Scheepmaker et al., 2016; Tonui et al., 2022). For example, *Pseudomonas* strains have been modified to overproduce antifungal metabolites, leading to better suppression of plant pathogens (Glandorf, 2019; Scheepmaker et al., 2016; Tonui et al., 2022). Genetically modified biocontrol agents could provide more targeted pest control while reducing reliance on chemical pesticides, aligning with goals for integrated pest management and sustainable agriculture (Scheepmaker et al., 2016).

However, the development and use of genetically modified biocontrol agents also raise important biosafety concerns that require careful consideration. One primary concern is the potential for unintended ecological impacts. While these biocontrol agents are designed to target specific pests, their release into complex ecosystems could have unforeseen consequences on non-target organisms or broader ecological processes. For instance, there are questions about whether genetically modified biocontrol agents could outcompete native microbial populations or disrupt existing ecological balances (Glandorf, 2019; Scheepmaker et al., 2016; Tonui et al., 2022).

Another significant concern is the potential for horizontal gene transfer. Genetically modified biocontrol agents carry modified genetic material, and there is a possibility that these genes could be transferred to other microorganisms in the environment. This could potentially lead to the spread of engineered traits beyond the intended biocontrol agent, with unknown consequences for microbial ecology and potentially human health (Glandorf, 2019; Scheepmaker et al., 2016; Tonui et al., 2022). The likelihood and potential impacts of such gene transfer events need to be thoroughly assessed as part of the risk evaluation process for genetically modified biocontrol agents.

## 8 Registration and commercialization of *Pseudomonas* spp. as biocontrol strains

In contrast to the established practice of biocontrol for insects, biocontrol methods targeting plant diseases represent a relatively recent development. This notwithstanding, the bacterium, *Agrobacterium radiobacter* strain K 84 was registered by the United States Environmental Protection Agency (EPA) as a means of controlling crown gall disease in 1979. A decade later, the first fungus, *Trichoderma harzianum* ATCC 20476, received EPA approval and registration for combating plant diseases.

To date, biocontrol agents represent <5% of the entire crop protection industry (Devaraj et al., 2019), despite their well-documented efficacy. The limited availability of licensed products for plant disease biocontrol is largely attributed to challenges in technology transfer, particularly in developing countries, where the economic potential of these agents is not completely recognized. Moreover, the mass production of promising microbial candidates for phytopathogen biocontrol often faces obstacles due to a lack of organism-specific research methods and the high costs associated with *in vivo* production and licensing, making them less competitive compared to existing chemical agents (Gehlot and Singh, 2018; Sundh and Goettel, 2013).

Ensuring effective deployment of biocontrol agents presents logistical challenges, including timing and density of application, as well as maintaining their presence in the target environment. Ecological concerns also arise from the introduction of non-native organisms, which may become invasive and disrupt local ecosystems. Despite promising results demonstrated in laboratory settings, many biocontrol agents have shown limited efficacy under real-world conditions (Backer et al., 2018; Capper and Higgins, 1993; Deacon, 1991; Mark et al., 2006), making them much less attractive to end users compared to chemical pesticides.

The release of GMOs, including engineered *Pseudomonas* strains, adds another layer of complexity to the registration and commercialization process. Regulatory frameworks for GMOs are typically more stringent than those for non-modified organisms, reflecting concerns about potential ecological impacts and gene transfer to wild populations. In the United States, for instance, the EPA regulates genetically engineered microbial pesticides under the Federal Insecticide, Fungicide, and Rodenticide Act (FIFRA) and the Toxic Substances Control Act (TSCA). These regulations require extensive risk assessments, including evaluations of genetic stability, potential for horizontal gene transfer, and impacts on non-target organisms (Angelo, 2023; Jones et al., 2024). The European Union has even more restrictive policies on GMOs, which mandate a case-by-case environmental risk assessment and post-market (Mizoguchi et al., 2024; Zimny, 2023).

The assessment of genetically modified biocontrol agents under existing regulations such as EC 1107/2009 (concerning the placing of plant protection products on the market) and Directive 2001/18/EC (on the deliberate release of GMOs into the environment) provides a foundation for evaluating their safety. However, these frameworks may need to be adapted or expanded to fully address the unique characteristics of living, replicating microbial agents (Glandorf, 2019; Scheepmaker et al., 2016; Tonui et al., 2022). This is particularly true for novel approaches like gene drive systems, which could potentially lead to the spread of engineered traits through wild populations more rapidly than traditional genetic modifications. These regulatory hurdles, while necessary for ensuring safety, can significantly delay the commercialization of genetically engineered *Pseudomonas* spp. biocontrol agents and increase development costs.

Public perception of GMOs significantly influences research trajectories in genetically modified biocontrol agent development. The reluctance to invest in technologies facing public skepticism has created a feedback loop, potentially impeding progress in sustainable pest management strategies (Lucht, 2015; Tonui et al., 2024). This phenomenon extends beyond funding constraints, affecting the entire research ecosystem.

The hesitancy to pursue genetically modified biocontrol research has broader implications for agricultural biotechnology. The overlap in expertise between genetically modified biocontrol development and other agricultural biotechnology applications suggests that reduced focus on gene modification research could lead to a general decline in capacity across the field (Qaim, 2020). This potential loss of human capital is particularly concerning in the context of early-career researchers, who may be dissuaded from specializing in gene modification-related fields due to perceived limited prospects (Merkley, 2020; Vanloqueren and Baret, 2017).

However, recent meta-analyses indicate a gradual shift in public attitudes toward GMOs in certain regions. It was reported an increasing trend of support for genetically modified foods in the United States over two decades, particularly among younger demographics and individuals with higher scientific literacy (Cui and Shoemaker, 2018). Similarly in Europe, an upward trend was observed in positive attitudes toward genetically modified crops between 2002 and 2019 (Woźniak et al., 2021). Multiple factors contribute to this attitudinal shift, including increased familiarity with GMOs, improved science communication, and growing recognition of biotechnology’s potential in addressing food security and environmental challenges (Fernbach et al., 2019).

Despite evolving public sentiment and scientific advancements, regulatory frameworks governing GMOs have remained largely static, creating a misalignment between current knowledge and regulatory approaches. Many jurisdictions maintain approval processes for GMOs established during periods of greater public skepticism (Eriksson et al., 2019). Additionally, current regulations often fail to account for advancements in genetic engineering techniques, such as CRISPR-Cas9, which allow for more precise genetic modifications. The regulatory focus on the process of genetic modification rather than the properties of the resulting organism may lead to over-regulation of genetically modified biocontrol agents that pose no greater risk than traditionally bred alternatives (Lassoued et al., 2019).

Current regulation and registration procedures, largely derived from chemical pesticide frameworks, do not adequately address the unique considerations of microbial biocontrol agents, contributing to their slow implementation in the field (Sundh and Goettel, 2013). As research on genetically modified biocontrol agents progresses, it will be crucial to conduct comprehensive, long-term studies on their ecological impacts. This should include assessments of their effects on soil microbial communities, potential impacts on beneficial insects and other non-target organisms, and their persistence in various environmental conditions. Such research will be essential not only for refining risk assessment procedures but also for building public trust in the technology (Glandorf, 2019; Scheepmaker et al., 2016; Tonui et al., 2022).

## 9 Conclusions and future perspectives

In the realm of sustainable agriculture, the use of microorganisms to combat plant diseases and enhance crop yield represents a promising alternative to chemical interventions. *Pseudomonas* spp., among others, have garnered attention for their potential as biocontrol agents due to their intricate interactions with plants and the environment; they are typically equipped with an arsenal of secondary metabolites and enzymes adept at engaging with host organisms and outcompeting adversaries. These bioactive molecules offer substantial benefits to crop plants by fostering plant growth and curbing disease incidence. Despite their resilience to environmental pressures, the efficacy of *Pseudomonas*-based biocontrol strategies hinges on a multifaceted interplay of factors, as elucidated in this review. While strides have been made to optimize delivery methods, mitigate stressors, and enhance soil, and root colonization, gaps persist in understanding the broader spectrum of influences, including agricultural practices, which shape plant microbiome dynamics and biocontrol efficacy.

To realize the full potential of biocontrol agents, comprehensive research efforts are imperative, with a focus on elucidating pre-existing signaling pathways between plants and microbes to bolster the colonization potential of desirable microbial strains. Moreover, investigations into the impact of candidate biocontrol strains on native microbial communities and ecosystem functions are paramount for deploying biocontrol agents with minimal non-target effects on a wider scale. The development of more consistent biocontrol strategies necessitates a holistic, combinatorial approach encompassing diverse aspects including delivery mechanisms, formulation enhancements, agricultural methodologies, and the intricate differences of plant-microbe symbioses. By embracing this integrated framework, it should be possible to realize effective, sustainable biocontrol solutions tailored to the complexities of modern agricultural landscapes.
